# Antimicrobial Activity against *Cronobacter* of Plant Extracts and Essential Oils in a Matrix of Bacterial Cellulose

**DOI:** 10.3390/polym16162316

**Published:** 2024-08-16

**Authors:** Lidia Stasiak-Różańska, Anna Berthold-Pluta, Tamara Aleksandrzak-Piekarczyk, Anna Koryszewska-Bagińska, Monika Garbowska

**Affiliations:** 1 Department of Food Technology and Assessment, Institute of Food Sciences, Warsaw University of Life Sciences—SGGW, Nowoursynowska St. 166, 02-787 Warsaw, Poland; 2 Institute of Biochemistry and Biophysics, Polish Academy of Sciences, Pawinskiego 5a, 02-106 Warsaw, Poland; 3 Department of Medical Biology, Medical University of Warsaw, Litewska 14/16, 00-575 Warsaw, Poland; akoryszewska@wum.edu.pl

**Keywords:** bacterial cellulose, plant extract, essential oil, antimicrobial activity, active packaging, *Cronobacter*

## Abstract

Bacterial cellulose (BC) is a biodegradable polymer resembling paper after being dried. It finds a growing number of applications in many branches of industry and in medicine. In the present study, BC was produced after *Gluconacetobacter hansenii* ATCC 23769 strain culture and used as a matrix for plant extracts (tulsi, brahmi, lemon, blackberry, nettle root, and nettle leave) and essential oils (cinnamon, sage, clove, mint, thyme, lemongrass, rosemary, lemon, anise, tea tree, lime, grapefruit, and tangerine), and the antimicrobial properties of these biomaterials was determined. The growth-inhibiting effects of plant extracts and essential oils combined with BC were analyzed against five *Cronobacter* species isolated from food matrix and two reference strains from the ATCC (513229 and 29544). Additional analyses were conducted for BC water activity and for its capability to absorb biologically active plant compounds. The cellulose matrix with a 50% extract from brahmi was found to effectively inhibit the growth of the selected *Cronobacter* strains. The other plant water extracts did not show any antimicrobial activity against the tested strains. It was demonstrated that BC soaked with thyme essential oil was characterized with the strongest antimicrobial activity in comparison to the other tested EOs. These study results indicate the feasibility of deploying BC impregnated with natural plant components as an active and environmentally-friendly packaging material.

## 1. Introduction

Bacterial cellulose (BC) is an exopolysaccharide composed of glucose monomers linked with β-1,4-glycosidic bonds. Its chemical formula is the same as that of the plant-derived one (C_6_H_10_O_5_)_n_ [1] and its nanofibrils are linked with hydrogen bridges. The crystallinity of BC ranges from 84% to 89% and, unlike plant-derived cellulose, it does not contain hemicellulose nor lignin. BC has a porous structure, which means that it can be easily soaked with many types of compounds, for instance, plant solutions. BC is resistant to stretching and is highly elastic [2,3]. Bacterial cellulose is produced extracellularly by acetic bacteria: *Gluconacetobacter* and *Komagataeibacter* genera [1,4]. 

BC is an interesting biomaterial featuring vast potential in the food industry. Owing to its GRAS status, it can be used as a food constituent and, simultaneously, as a source of dietary fiber [5]. It has for years been deployed to produce a natural beverage based on fermented tea–kombucha, and a coconut dessert called “nata de coco” popular in the, a.o., Philippines. Its implementation potential in the industry stems from, a.o., its strong mechanical resilience, high water vapor permeability, and elasticity [1]. Its properties are exploited in the food industry to, e.g., thicken foods or beverages. It may also be applied as a food packaging, including, e.g., active packaging which enables extending a food product’s shelf-life [5]. BC biodegradability allows for classifying this polymer as an alternative to food packaging made from plastics [3]. It was demonstrated that BC with xanthan and CeO_2_ nanocomposites was an effective coating material for antimicrobial food packaging applications, for example, bread packaging [6]. To improve the properties of BC as an active packaging material, various techniques can be used to modify and functionalize this biopolymer, as well as combine BC with other biological agents. Using these combined methods it is possible to obtain food packaging with properties tailored to individual needs [7].

Essential oils (EOs) are natural volatile compounds found in plant organisms, including edible plants, herbs, and plants with therapeutic properties [8]. They are sourced from various morphological parts of plants, i.e., flowers, buds, leaves, bark, fruits, and tubers [9], and impart characteristic flavors and aromas to them, and can also affect their taste values. Essential oils are produced as secondary plant metabolites and are secreted especially upon plant exposure to various stress factors, like attack of pathogens or adverse changes in natural environment conditions. They may also play a meaningful role in plant reproduction by attracting insects [8,10]. 

The chemical composition of essential oils varies depending on the species of plant they derive from, the place of cultivation, and the climatic conditions. Their properties stem from the presence of various functional groups, chemical configuration and synergistic interactions between their components. Their major compounds include terpenes and their derivatives (terpenoids), as well as aromatic and aliphatic compounds [8]. The oils and their components are used as preservatives and agents imparting flavor to food [8]. They exhibit anti-inflammatory properties as well as antimicrobial activity. The antibacterial effect of essential oils is due to their multiple active compounds that affect bacterial cells, like, e.g., phenolic compounds, including especially carvacrol, thymol, and eugenol [11,12]. Their hydrophobic nature increases the permeability of the bacterial cell membrane. 

Bacteria of the genus *Cronobacter* are Gram-negative, relatively anaerobic rods with dimensions of 3 μm × 1 μm [13]. They are motile and tolerate low pH values, high temperatures and low water activity in the environment [14]. They can grow at temperatures from ca. 5 °C to 47 °C and survive the spray drying process [15]. *Cronobacter* bacteria produce biofilms, which determines, among others, their resistance to disinfectants, antibiotics, and water shortage in the environment [14]. The *C. malonaticus, C. sakazakii*, and *C. turicensis* species have been isolated from clinical cases of neonatal infections as the only ones from the genus *Cronobacter* [15]. Among these, the greatest number of food poisonings in humans has been caused by *C. sakazakii* infection. These bacteria pose a threat to the health of newborns and infants with low birth weight, as well as persons with compromised immunity [16]. They induce diseases such as sepsis, bacteremia, meningitis, and necrotizing enterocolitis. The mortality rate due to *Cronobacter* spp. infections is as high as 80%. The most common source of infection with *Cronobacter* spp. is, i.a., milk formulas for infants. There are several mechanisms of *Cronobacter* spp. bacteria virulence, like protein A (OmpA), which is an element of the outer membrane of bacterial cells and makes it possible to attack, for example, intestinal epithelial cells. *Cronobacter* spp. bacteria also produce enterotoxin, the role of which may be related to the disruption of the blood–brain barrier. Their virulence is also influenced by the adhesion of their cells to the walls of the cerebral vessels and the intestinal epithelium [14].

Active packaging is a type of packaging containing additives whose function is to maintain the high quality of a food product or extend its shelf life. They contain active agents that can react with the environment surrounding the product and enter into reactions with it. Currently, active packaging has gained in popularity in response to the needs of the growing global population and the urge to develop new, biodegradable packaging that will reduce food waste while protecting the environment. Active packaging is used for various types of food, including, e.g., those containing essential oils, which can be used to pack fish and meat [17], and also in the cosmetic and pharmaceutical industries. Antimicrobial components used in active packaging inhibit the growth of pathogenic microflora and food-spoiling microflora. Many compounds may exhibit antimicrobial activity in food products, e.g., essential oils (e.g., oregano oil), plant extracts (e.g., green tea extract), metal oxides (e.g., zinc oxides), peptides (e.g., nisin) or some enzymes (e.g., lysozyme, lactoferrin) and even ions (e.g., silver) [17]. Antimicrobial components are incorporated into active packaging by covering or impregnating the carrier material with them [18,19]. The use of essential oils as components in the active packaging of food products enables their controlled release in a manner acceptable to consumers sensitive to their aroma and those who are opponents of food preservatives. In order to test the antimicrobial properties of active packaging impregnated with essential oils and the oils themselves, for example, the growth inhibition zones of the tested microorganisms are measured using the disc diffusion method [20].

The research goal was to develop a prototype biodegradable material based on bacterial cellulose as a proposition of packaging intended for contact with food. 

## 2. Materials and Methods

### 2.1. Test Strains

Two reference *Cronobacter* strains: *C. muytjensii* ATCC 51329 and *C. sakazakii* ATCC 29544, both from the American Type Culture Collection, and isolates from the plant matrix [21]: *C. sakazakii lv27*, *C. malonaticus lv31*, *C. condimenti s37*, *C. muytiensi s50*, and *C. turicensis lv53*, were used in this study.

### 2.2. Plant Extracts and Essential Oils

This study was conducted with 6 aqueous extracts of chosen plants (20% or 50% mass/volume) and 13 different essential oils.

Aqueous extracts used in this study (Applied Biochemistry Sp. z o.o., Warsaw, Poland):

- tulsi (*Ocimum sanctum*)
- brahmi (*Bacopa monnieri*)
- lemon (*Citrus limon*)
- blackberry (*Rubus* L.)
- nettle root (*Urtica dioica* L.–*radix*)
- nettle leaves (*Urtica dioica* L.–*folium*) (Applied Biochemistry Sp. z o.o., Poland)

Essential oils used in this study (BAMER, Poland, steam distillation, composition declared by the manufacturer):

- sage from the sage bush (*Salvia shrub*) composed of: litronellol, geraniol, limonene, citral, and linalool;
- clove from buds (*Eugenia caryophyllus*) composed of: eugenol and isoeugenol;
- cinnamon from cinnamon tree bark (*Cinnamomum zeylanicum*) composed of: cinnamal, linalool, eugenol, d-limonene, benzyl benzoate, coumarin, and cinnamyl alcohol;
- mint from mint leaves (*Mentha* L.) composed of: l-menthol, menthone, menthyl acetate, limonene, pulegone, and carvone;
- thyme from thyme leaves (*Thymus vulgaris*) composed of: linalool, limonene, citral, and geraniol;
- lemongrass from East Indian lemongrass (*Cymbopogon flexuosus*) composed of: citral, geraniol, linalool, limonene, and citronellol;
- rosemary from rosemary leaves/stems (*Rosmarinus officinalis*) composed of: limonene and linalool;
- lemon from lemon peel (*Citrus limonum*) composed of: limonene, citral, linalool, citronellol, and geraniol;
- anise from star anise (*Illicium verum*) composed of: limonene, linalool, and geraniol;
- tea tree (*Melaleuca alternifolia*) composed of: limonene, linalool, and geraniol;
- lime (*Citrus aurantifolia*) composed of: limonene, citral, geraniol, linalool, and citronellol;
- grapefruit (*Citrus paradisi*) composed of: limonene, citral, linalool, geraniol, eugenol, benzyl benzoate, and citronellol;
- tangerine (*Citrus reticulata* Blanco) composed of: limonene and linalool.

Powdered, ready-made extracts, obtained directly from the producer, were dissolved in sterile distilled water at 25 °C. Concentrations of 20% and 50% (mass/volume) of the extract in water were used for research. Aqueous plant extracts were used immediately after preparation.

### 2.3. Preparation of Discs from Bacterial Cellulose

BC was obtained from the culture of the *Gluconacetobacter hansenii* ATCC 23769 strain, grown at 28 °C for 10 days in medium with the following composition [gL^−1^]: yeast extract 0.5; peptone 0.3; and mannitol 2.5, with pH 5.75. All of the media components were obtained from BTL Poland S.A., and the media was sterilized at 121 °C for 15 min.

**Figure 1:**
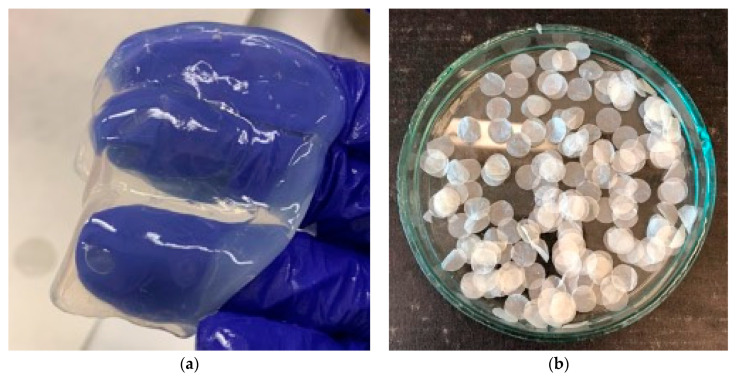
(**a**) Purified BC before drying; (**b**) discs cut out of dried BC.

BC was harvested from the surface of the medium and purified by rinsing in distilled water and incubating in 0.1 M NaOH, at 95 °C for 60 min. Afterward, it was neutralized with acetic acid to pH 7.0. The cellulose films were dried at 22 °C in a fume cupboard for 24 h. Then, discs 0.8 cm in diameter were cut from the 0.05 mm thick dried bacterial cellulose using a sterile cork borer, put into glass vials and sterilized at 121 °C for 15 min. Figure 1 shows the purified wet bacterial cellulose and cellulose discs obtained after cellulose drying.

### 2.4. Microbiological Media

*Cronobacter* strains were cultured in a tryptone–soy medium (BioMaxima S.A., Lublin, Poland) with the following composition [gL^−1^]: pancreatin hydrolysate of casein 3.4; soy peptone 0.6; sodium chloride 1; dipotassium hydrogen phosphate 0.5; glucose monohydrate 0.5; if necessary, agar 3; pH 7.0. The medium was sterilized at 121 °C for 15 min.

### 2.5. Determination of Water Activity in Dry Bacterial Cellulose

The water activity was determined in the dried BC with the HygroPalm 23-AW-A device (Rotronic, Bassersdorf, Switzerland), according to the manufacturer’s instructions.

### 2.6. Determination of the Absorption Capacity of Essential Oils by Bacterial Cellulose

Weight measurements were made for dried and sterilized bacterial cellulose discs as well as bacterial cellulose discs impregnated with plant extract, essential oils or sterile water (24 h after disc impregnation). The results obtained allowed for determining the average weight of the bacterial cellulose disc impregnated with a given substance and the average weight of the dry bacterial cellulose disc. The absorption capacity of the essential oils by bacterial cellulose was determined from the formula:

(%) = [(*M_i_*– *M_d_*)/*M_i_*] × 100%

where: *M_i_*—mass of the bacterial cellulose disc impregnated with essential oil or sterile water, and *M_d_*—mass of the dry bacterial cellulose disc [22].

### 2.7. Disc Diffusion Method

A respective *Cronobacter* isolate from the suspension of a single colony with a density of 0.5 McFarland was sown on a dish with tryptone–soy medium, then a CB disc impregnated (24 h at 22 °C) with an appropriate biologically active compound or water (control) was placed on the dish, which was then tightly wrapped with parafilm and incubated at a temperature of 33 °C for 24 h. Afterward, the diameter of the growth inhibition zones of the tested strains was measured using an electronic caliper (SW-DC-150N, PRO Sp. z o.o., Bielsko-Biała, Poland). Each analysis of antimicrobial activity was performed in five independent replicates.

### 2.8. Statistical Analysis

The significance of differences in the diameters of the growth inhibition zones was investigated for various isolates and active ingredients used in the matrix (extracts and oils). The results obtained were subjected to a statistical analysis using Statistica, version 13 software (TIBCO Software Inc., Kraków, Poland, 2017). One-way (ANOVA) analysis of variance was utilized. Tukey’s test was used to compare the significance of differences between mean values at a significance level of α = 0.05.

## 3. Results and Discussion

### 3.1. Water Activity of Bacterial Cellulose

Water activity is an important parameter influencing food quality. Its optimal range for the development of most microorganisms is 0.990–0.995. The minimum value of water activity needed for the growth of microorganisms depends on their genus, e.g., 0.9 for most bacteria, 0.8 for yeast, and 0.7 for molds [23]. Measurements of the water activity of the material used in active packaging allow for determining whether the microorganisms will be able to survive in a given environment.

The water activity of dry and purified BC was measured in order to characterize this biopolymer as a material suitable for the production of active packaging. In the present study, the mean a_w_ measured at a temperature of 20 °C for the bacterial cellulose samples used in the disc diffusion method was 0.43. This value suggests that BC could be a fine material for producing packaging, because a low water activity would effectively suppress or even completely inhibit the development of undesirable microflora in the packed product. Biocellulose is a biopolymer that can be applied as an edible packaging. When used for the production of packaging along with natural plant extracts and essential oils, it can extend the shelf-life of food products [24,25]. Garavito et al. [26] assessed the possibility of using selected films as active packaging. The water activity of the dry films containing guar gum and soy protein averaged 0.51, which supported the conclusion that the tested films could be a potential base material for the production of active packaging, providing conditions unfavorable to the development of most microorganisms.

### 3.2. Antimicrobial Activity of Bacterial Cellulose Impregnated with Plant Extracts against Cronbacter Strains

**Figure 2:**
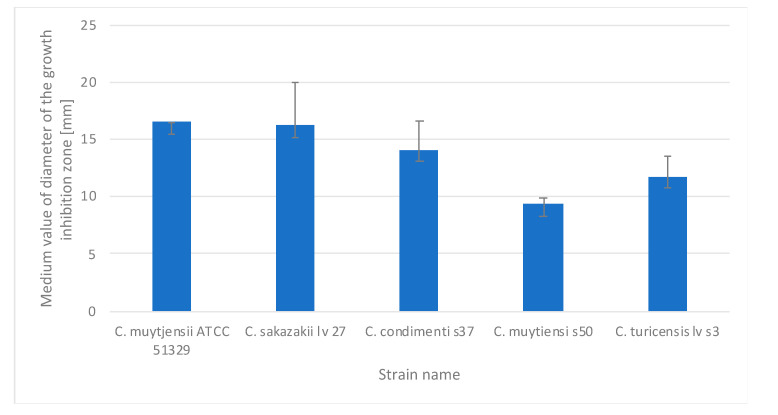
Diameters of zones of growth inhibition (with standard deviation) of the tested *Cronobacter* strains by BC discs soaked with 50% aqueous brahmi extract.

Analyses of the antimicrobial activity of BC impregnated with 20% aqueous extracts of tulsi, brahmi, blackberry, lemon, nettle root, and nettle leaves against *Cronobacter* bacteria showed no growth inhibition zones. Probably, the 20% concentration of these extracts was insufficient to inhibit the growth of the tested strains; therefore, 50% solutions of these extracts were prepared and analyzed in the next stage of the study. BC impregnated with 50% tulsi, lemon, blackberry, root and nettle leaf extracts also showed no antimicrobial activity against the tested *Cronobacter* strains. In contrast, the 50% brahmi extract showed antimicrobial activity against five out of the seven strains tested (Figure 2).

BC impregnated with a 50% aqueous solution of brahmi extract showed the greatest inhibitory effect against the reference strain *C. muytjensii* ATCC 51 329, where the diameter of the inhibition zone was 16.48 mm, and against the isolate of *C. sakazakii lv27*, where the diameter of the inhibition zone was 16.19 mm. Bacterial cellulose impregnated with 50% brahmi extract showed the lowest inhibitory effect against the *C. muytjensii s50* isolate (9.29 mm). The 50% aqueous extract of brahmi plant in combination with BC had no inhibitory effect on the growth of *C. malonaticus lv31* and *C. sakazakii* ATCC 29 544 isolates.

The brahmi extract is recognized in Ayurveda as a medicinal substance and may exhibit antimicrobial activity against selected strains due to the presence of *n*-butanol [27,28]; however, many studies do not seem to confirm any antimicrobial effect of its aqueous extract. The disc diffusion method with aqueous brahmi extracts as well as with ethanol, diethyl ether, and ethyl acetate brahmi extracts demonstrated an inhibitory effect of diethyl ether extract against *Staphylococcus aureus*. In addition, an ethyl acetate brahmi extract showed antimicrobial properties against *E. coli.* The cited studies did not demonstrate any antimicrobial effect of aqueous brahmi extracts against *S. aureus* and *E. coli* strains [27]. Kalyani [29] and Sampathkumar et al. [30], also made similar observations, stating that aqueous brahmi extracts did not exhibit antimicrobial activity against *S. aureus* and *Proteus vulgaris*, *Salmonella enteritidis*, or *Pseudomonas aeruginosa*, possibly due to the loss of some active compounds during the sample extraction process. Both the cited studies and our results may suggest that the antimicrobial activities of aqueous brahmi extracts are correlated with the genera, species or even strain of bacteria.

### 3.3. Antimicrobial Activity of Essential Oils on the Matrix with BC against Cronobacter

The diameters of the zones of inhibition of the *Cronobacter* strains induced by BC impregnated with selected essential oils and sterile water (control) are presented in Table 1. Based on the results obtained, including the dimensions of the bacterial cellulose disc (0.8 cm in diameter) and the method of analyzing the results described in Nagmetova et al. [22], the following criteria were adopted to determine the antimicrobial activity of BC impregnated with essential oils: growth inhibition zone of >20 mm—strong antimicrobial activity, growth inhibition zone of 12–20 mm—moderate antimicrobial activity, and growth inhibition zone of <12 mm—no antimicrobial activity.

Strong antimicrobial activity of BC with cinnamon EO was demonstrated, not only against the ATCC 51329 strain, but also against *C. malonaticus lv31* (32.10 ± 0.87 mm). Similar diameters of the growth inhibition zones were observed for *C. condimenti s37* (29.05 ± 1.78 mm) and the *C. sakazakii* ATCC 29 544 (28.46 ± 1.27 mm). Cinnamon oil shows a strong antibacterial effect due to the presence of cinnamaldehyde, the action of which contributes to the deformation and likely disruption of the cell membrane of Gram-negative bacteria [31]. Cinnamon essential oil also contains eugenol as well as benzaldehyde, cinnamyl acetate, dihydrocinnamic aldehyde, and cuminol. Using the disc diffusion method, Berthold-Pluta et al. [32] showed that the zone of *Cronobacter* growth inhibition by plant cellulose impregnated with cinnamon oil was >30 mm. The strong antimicrobial effect of cinnamon essential oil delivered from a plant cellulose disc against other Gram-positive bacteria (*E. coli* and *P. aeruginosa*) as well as against Gram-positive bacteria (*S. aureus* and *S. epidermidis*) has been confirmed and described in numerous scientific publications [31,33,34].

Sage essential oil turned out to be a weak inhibitor of the growth of *Cronobacter* genus bacteria. BC with sage EO caused moderate inhibitory activity (13.21 ± 2.29 mm) against *C. condimenti s37*. In the case of other tested strains, the diameter of the growth inhibition zones was <12 mm, indicating no inhibitory effect. It can, therefore, be concluded that BC impregnated with sage EO was ineffective in preventing the growth of *Cronobacter* bacteria (except for *C. condimenti s37*), although according to literature data, this essential oil was found capable of inhibiting the growth of selected Gram-negative bacteria, e.g., *E. coli*, *S. typhimurium*, *Klebsiella pneumonia*, and *Pseudomonas aeruginosa* as well as Gram-positive ones, including *S. epidermidis*. Nevertheless, it exhibits an antimicrobial effect only at high concentrations, especially when used against Gram-negative bacteria [35,36,37].

BC impregnated with clove essential oil showed a strong inhibitory effect against reference *C. muytjensi* ATCC 51329 (21.50 ± 1.55 mm) and a moderate inhibitory effect against the rest of the tested strains. In its case, the diameters of the growth inhibition zones of the tested strains ranged from 12.23 ± 0.58 mm (for *C. turicensis lv53*) to 21.50 ± 1.55 mm (for ATCC 51329). A similar inhibitory effect of BC with clove EO was observed for the isolates of *C. muytjensii s50* (20.40 ± 3.40 mm) and *C. condimenti s37* (19.73 ± 1.69 mm) as well as *C. sakazakii lv27* and *C. malonaticus lv31* (ca. 15.95 mm). The major compound of clove essential oil is carvacrol, accounting for 77% of its total composition and causing its moderate inhibitory effect [38]. The antibacterial activity of clove EO against pathogenic strains, including A. baumanni, E. faecalis, and P. aeruginosa, was demonstrated by Baydaa et al. [39]. In the case of *C. jejuni* Gram-negative bacteria, only changes in their cell morphology were shown, whereas their growth inhibition was not confirmed [40]. The antimicrobial activity of this oil has also been demonstrated against Gram-positive bacteria (*L. monocytogenes* and *B. cereus*) [41].

The diameters of the growth inhibition zones of *Cronobacter* bacteria caused by BC impregnated with mint essential oil are shown in Table 1. The most susceptible to the action of BC with mint EO turned out to be the following strains: ATCC 51 329—with a growth inhibition zone of 15.61 ± 2.32 mm, and *C. condimenti s37*—with a growth inhibition zone of 14.92 ± 2.33 mm. The major compound of this EO is sinistral menthol and this EO’s antimicrobial activity is owed to this very compound [42]. It can account for up to 80% of the total EO composition, next to menthyl acetate (approx. 22%), menthone (approx. 2.5%) and other compounds. In their study of the effect of essential oils on the quality of juices, De Sousa Guedes et al. [43] demonstrated that mint essential oil may inhibit the growth of *E. coli* and *S. enteritidis*. Research entailing the disc diffusion method with plant cellulose impregnated with mint essential oil showed that the diameter of *E. coli* growth inhibition may reach about 18 mm [44]. Analogous studies have shown that growth inhibition zones caused by the antimicrobial properties of mint EO may be as large as approx. 38 mm for *S. aureus* [45], and also 45.5 mm and 19.6 mm for *P. vulgaris* and *L. plantarum* bacteria, respectively [46].

Thyme essential oil on the BC matrix showed a moderate and strong capability for inhibiting the growth of all tested bacterial strains, extending from the growth inhibition zone of 17.14 ± 2.15 mm in the case of *C. turicensis lv53*, which showed the greatest resistance to its antimicrobial effect, to complete growth inhibition in the case of the *C. sakazakii lv27* isolate. A strong antimicrobial effect of BC soaked with thyme EO was shown against reference strain ATCC 51329. For this strain, the diameter of the inhibition zone was 44.37 ± 3.33 mm. Another strong and similar effect was observed for *C. malonaticus lv31* and *C. condimenti s37*. Our previous research [32] demonstrated that BC impregnation with thyme EO allowed for obtaining a biomaterial with strong antimicrobial properties. A similar study [47], with the use of BC impregnated with thyme EO, showed a significant inhibition of *P. aeruginosa* and *S. aureus* growth.

The lemongrass EO used in this study contained citral, geraniol, linalool, limonene, and citronellol. BC with lemongrass EO showed moderate antimicrobial activity against *C. muytjensii* ATCC 51329, *C. sakazakii* ATCC 29544, *C. sakazakii lv27*, and *C. malonaticus lv31*. The lemongrass essential oil containing, a.o., linalool, geraniol, neral and geranial, exhibited antimicrobial properties against other Gram-negative bacteria, including *Acinetobacter baumannii* [48]. In turn, lemongrass essential oil containing, a.o., neral and geranial, showed an inhibitory effect on *B. cereus* growth, which suggests that it may also inhibit the growth of other Gram-positive bacteria [49].

The diameters of the growth inhibition zones caused by BC impregnated with rosemary essential oil ranged from 9.47 ± 0.08 mm (*C. sakazakii lv27*) to 18.20 ± 1.01 mm (*C. malonaticus lv31*). These results confirming the moderate antimicrobial activity of BC with this EO were obtained for *C. malonaticus lv31*, *C. condimenti s37*, *C. muytjensii s50*, and *C. turicensis lv53*, while it was proved ineffective against ATCC 51329, ATCC 29544 and *C. sakazaki lv27*. The antimicrobial properties of rosemary essential oil are due mainly to phenolic diterpenes, rosmarinic and carnosic acids, as well as carnosol. In turn, its chemical composition is largely dependent on the geographical region the plant derives from, the growing climate, the method of essential oil production, and the plant genotype [50]. A study by Chao et al. [49] did not confirm the antimicrobial effect of rosemary essential oil against *B. cereus* bacteria, indicating the selective inhibitory effect of this EO and the relationship between its composition and antimicrobial properties.

Tea tree essential oil combined with BC showed a moderate inhibitory effect on the growth of *C. muytjensi s50* and *C. turicensis lv53* isolates, for which the diameters of the growth inhibition zones were 12.59 ± 2.53 mm and 19.80 ± 2.72 mm, respectively. The remaining strains tested were more resistant to BC with tea tree EO, as evidenced by their growth inhibition zone diameters not exceeding 12 mm. Tea tree EO is usually composed of various monoterpenes (terpinene-4-ol, terpinolene, p-cymene, α-pinene, γ-terpinene, and 1,8-cineole), sesquiterpenes, and the corresponding alcohols (monoterpene and alcohol-terpineol). Its major compounds are methylegenol and phenylpropanoids [51]. Previous studies confirmed that this essential oil exhibited antimicrobial potential against, i.a., *L. monocytogenes* and *E. coli* [52], which was probably due to the presence of hydroxyl groups eliciting bacteriostatic and bactericidal effects. The hydrophobic properties of tea tree essential oil facilitate the destabilization and degradation of lipids present in the bacterial cell membrane, causing the loss of cellular components and ions, consequently leading to cell death [53]. Król et al. [54] demonstrated the inhibitory effect of tea tree essential oil against *Propionibacterium acne*, *Serratia marcescens*, *Pseudomonas aeruginosa*, *Klebsiella pneumoniae*, *Streptococcus mutans*, *Staphylococcus aureus*, *S. epidermidis*, *S. haemolyticus*, *Streptococcus pyogenes*, and *S. salivarioniae* bacteria.

It should be emphasized that without the addition of active substances, BC did not show any antimicrobial properties, which was confirmed both by our study results (control sample) and by findings of other scientists [55].

Due to their documented antimicrobial activity, essential oils are currently spurring great interest among producers of, a.o., food, cosmetics and pharmaceuticals. Most of the essential oils have GRAS status, which enables their use in contact with food [9]. Most essential oils show greater antimicrobial activity against Gram-positive than Gram-negative bacteria [56], most likely due to the presence of outer membrane proteins or lipopolysaccharides in the latter. 

**Figure 3:**
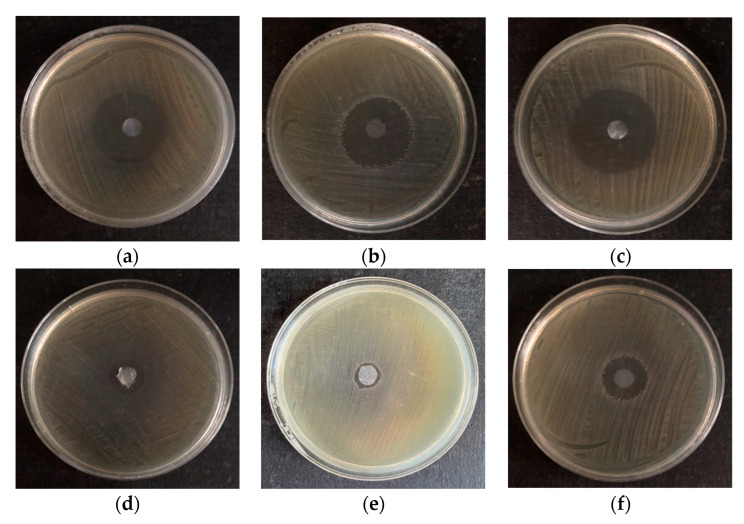
Chosen Petri dishes showing the biggest (first line) and the lowest (second line) inhibition zones of BC soaked with EO against *Cronobacter* strains; (**a**)—BC+cinnamon against *C. malonaticus lv31*; (**b**)—BC+thyme against *C. muytjensi s50*; (**c**)—BC+cinnamon against *C. muytjensi s50*; (**d**)—BC+clove against *C. malonaticus lv31*; (**e**)—BC+sage against ATCC 51329; (**f**)—BC+lemongrass against *C. muytjensi s50*.

Pictures of chosen Petri dishes with visible zones of growth inhibition are presented in Figure 3.

### 3.4. Assessment of the Absorption Capacity of Extracts and Essential Oils by Bacterial Cellulose

Bacterial cellulose can absorb up to 200 times more water than its dry weight [57]. Its water absorption capacity is due to the presence of many hydroxyl groups and the action of capillary forces [58]. The possibility of liquid absorption by BC allows this biomaterial to be used in active packaging as a moisture absorber and/or carrier of biologically active compounds. Bacterial cellulose has a greater water-holding capacity compared to plant-derived cellulose. The water absorption capacity of BC may result from differences in the arrangement of cellulose fibers that make up its structure, its surface area to the mass unit ratio, and its porosity. Generally, the larger the surface area and the pore size of bacterial cellulose, the greater its water absorption capacity. The value of this parameter may also be influenced by the substrate used to produce the BC, as well as by the method of its production [59].

**Figure 4:**
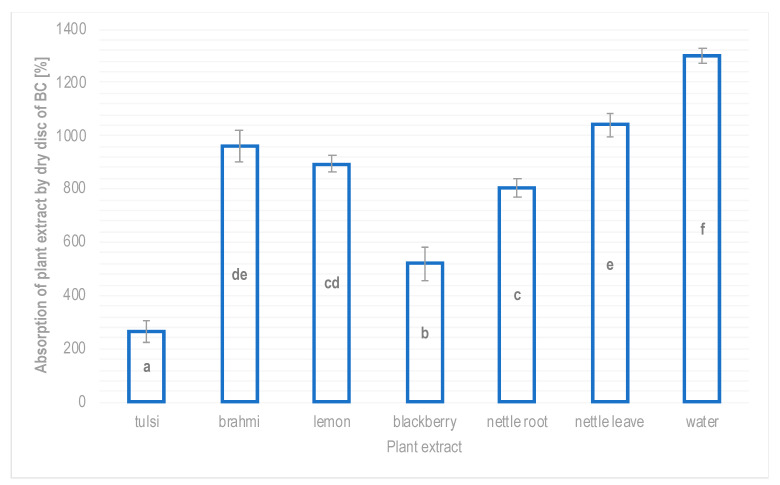
Absorption of aqueous solutions of 50% plant extracts by BC discs obtained from *Gluconacetobacter hansenii* ATCC 23769 (mean values and standard deviations, a–f—homogenous groups, *p* ≤ 0.05, n = 3).

**Figure 5:**
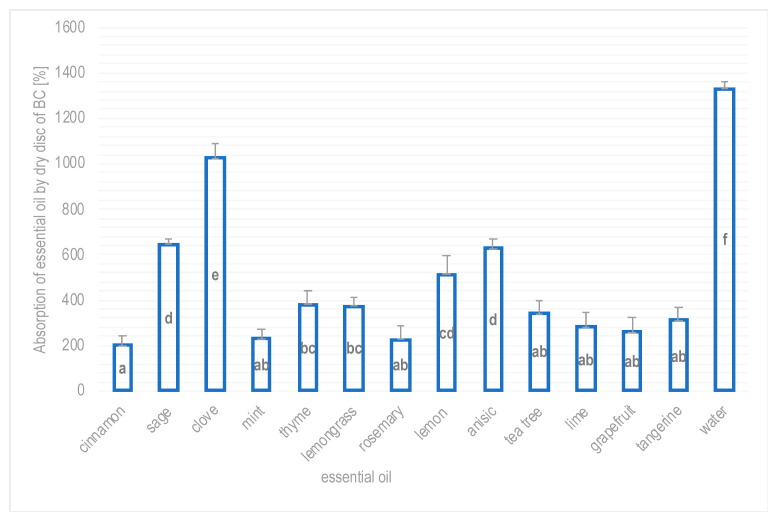
Absorption of selected essential oils by BC discs obtained from *Gluconacetobacter hansenii* ATCC 23769 (mean values and standard deviations, a–f—homogenous groups, *p* ≤ 0.05, n = 3).

The absorption capacity of plant extracts and essential oils was measured in this study and the respective results are presented in Figure 4 and Figure 5.

The dry BC disc after absorbing water weighed 14 times more than before soaking, so the absorption in this case was 1300%. In the case of 50% plant extracts, BC showed the lowest absorption for the aqueous tulsi extract (approx. 265%), which means that the soaked disc weight was approximately 3.65 times more than the dry disc. Biocellulose impregnated with an aqueous blackberry extract showed an absorption capacity of 519% (weight of the soaked disc was over six times more than the dry disc weight). The absorption capacity of the aqueous extracts from lemon and nettle root by the BC was similar, i.e., 894% and 805%, respectively, and slightly higher in the case of the aqueous extract from brahmi (961%). The dry disc of BC showed the highest holding capacity for nettle leaf extract, which was almost 11.5 times more in comparison to the unsoaked disc.

BC showed the greatest ability to absorb clove essential oil. In this case, the dry disc of BC took over 11 times more clove essential oil by weight than before soaking. Its slightly lower, but still significant, absorption capacity was demonstrated against sage (645%) and anise (627%) essential oils, and slightly lower against lemon essential oil (514%). The absorption capacity of the remaining essential oils by BC ranged from 200% (for cinnamon oil) to 382% (for thyme oil).

Literature data on the absorption capacity of essential oils by BC are limited. Among the available publications where this parameter was tested for materials impregnated with essential oils, measurements were made several times at intervals, and the length of the entire analysis was shorter than in the present study. Our previous study [22] investigated the absorption capacity of oregano essential oil by BC obtained from the *G. hansenii* ATCC 23579 strain. Measurements were made at 10 min intervals, starting from the 10 min soaking of bacterial cellulose. The results obtained indicated approx. 40% absorption of this EO by BC. The results obtained in the present study showed a much higher absorption capacity of essential oils by BC, which could be due to the time of the measurement, because the impregnated BC discs were weighed 24 h after immersion in the tested essential oils. Junka et al. [47] determined the water absorption capacity of bacterial cellulose at 68%. They also measured the absorption capacity of thyme oil by bacterial cellulose, which ranged from 30 to 40%. The values of the absorption capacity of essential oils by bacterial cellulose obtained in this study were greater than those presented by Junka et al. [47]. This difference could be due to the different method used by these authors for draining cellulose impregnated with EOs, because they used centrifugation at a speed of 2000 rpm for 5 min. The second difference was that they made the measurements every 5 min within a period of 20 min.

Both in the studies by Junka et al. [47] and Nagmetova et al. [22], whose main goal was to test the antimicrobial activity of BC impregnated with essential oils, it was found that the essential oils absorbed by BC inhibited the growth of the tested bacterial strains, which is confirmed by the research presented in this paper. The values of the absorption capacity of essential oils by BC obtained in these studies could be due to, e.g., different drying conditions of bacterial cellulose before its impregnation with the tested substances. The process of water evaporation during drying may depend, among others, on the temperature and humidity of the environment. The level of air saturation with water vapor may influence the transformation of water that has evaporated from the surface of bacterial cellulose into that which is bound in its structure. Ambient humidity could also prove to be a factor influencing the absorption of essential oil by bacterial cellulose.

## 4. Summary

The aim of this study was to investigate whether a biodegradable polymer (bacterial cellulose) enriched with natural active substances (plant extracts and essential oils) could serve as a matrix for the development of active packaging. The BC obtained in this study and then impregnated with selected natural biologically active substances was tested against *Cronobacter* strains isolated from food matrix. The greatest ability to inhibit the development of the studied strains was demonstrated for BC impregnated with thyme oil. The inhibitory effect of BC with extracts/oils varied depending on both the type of extract used and the strain tested.

The study results clearly suggest that the BC soaked with active plant substances showed antimicrobial potential and that research aimed at developing an innovative ecological active food packaging based on BC enriched with biologically active agents should be continued. One avenue of research worth exploring is the possibility of combining several essential oils in a single sample of BC, which could possibly enhance the antimicrobial activity of the impregnated BC. 

## Figures and Tables

**Table 1:** Zones of bacterial growth inhibition by bacterial cellulose soaked with selected essential oils. Mean and sd values from five independent replicates.

Bacterial Strain	Inhibition Zone [mm] ± SD Caused by the Presence of a Given Essential Oil													
	Oil													Control
	Cinnamon	Sage	Clove	Mint	Thyme	Lemongrass	Rosemary	Lemon	Anisic	Tea Tree	Lime	Grapefruit	Tangerine	
*C. muytjensii* ATCC 51329	34.62 e ± 1.51	10.05 b ± 0.81	21.50 c ± 1.55	15.61 a ± 2.32	44.37 c ± 3.30	18.81 c ± 1.27	11.82 ab ± 0.13	0.00 a	10.32 b ± 0.24	8.69 a ± 0.60	9.10 a ± 1.14	0.00 a	0.00 a	0.00 a
*C. sakazakii* ATCC 29544	28.46 c ± 1.27	9.88 b ± 0.15	16.73 b ± 0.31	13.04 a ± 1.06	21.80 a ± 1.03	12.90 ab ± 0.57	11.02 ab ± 0.96	0.00 a	0.00 a	10.18 a ± 0.71	9.27 ab ± 1.15	0.00 a	0.00 a	0.00 a
*C. sakazakii* lv27	24.17 b ± 1.02	0.00 a	15.95 ab ± 0.67	11.89 a ± 0.88	90.53 d ± 1.29	13.90 b ± 0.88	9.47 a ± 0.08	0.00 a	0.00 a	11.05 a ± 1.69	10.99 ab ± 1.55	0.00 a	0.00 a	0.00 a
*C. malonaticus* lv31	32.10 de ± 0.87	0.00 a	15.94 ab ± 0.56	13.91 a ± 2.02	30.61 b ± 1.99	12.27 ab ± 0.64	18.20 c ± 1.01	0.00 a	0.00 a	9.98 a ± 1.34	13.00 b ± 2.00	0.00 a	0.00 a	0.00 a
*C. condimenti* s37	29.05 cd ± 1.78	13.21 c ± 2.29	19.73 bc ± 1.69	14.92 a ± 2.33	30.87 b ± 0.28	11.52 a ± 0.19	14.10 abc ± 3.12	10.35 b ± 0.27	0.00 a	11.97 a ± 2.02	9.24 ab ± 1.74	0.00 a	0.00 a	0.00 a
*C. muytjensii* s50	24.19 b ± 1.27	9.72 b ± 0.55	20.40 bc ± 3.40	12.31 a ± 1.90	29.59 b ± 1.71	11.62 a ± 0.21	16.72 c ± 2.54	0.00 a	0.00 a	12.59 a ± 2.53	9.11 a ± 1.00	0.00 a	0.00 a	0.00 a
*C. turicensis* lv53	9.51 a ± 0.52	11.26 c ± 0.50	12.23 a ± 0.58	12.89 a ± 1.13	17.14 a ± 2.15	11.37 a ± 0.09	14.19 bc ± 1.25	0.00 a	0.00 a	19.80 b ± 2.72	8.44 a ± 0.51	0.00 a	0.00 a	0.00 a

Means ± standard deviations: a–e—means with different letters in column for a 1 oil are significantly different (*p* < 0.05, n = 3). Colors mean: green—strong antimicrobial activity (>20 mm inhibition zone); yellow—moderate antimicrobial activity (12–20 mm inhibition zone); red—no antimicrobial activity (<12 mm inhibition zone).

## Data Availability

All data are available in the article.
